# Limiting treatment and shortening of life: data from a cross-sectional survey in Germany on frequencies, determinants and patients’ involvement

**DOI:** 10.1186/s12904-016-0176-6

**Published:** 2017-01-17

**Authors:** Birte Malena Dahmen, Jochen Vollmann, Stephan Nadolny, Jan Schildmann

**Affiliations:** 1 Institute for Medical Ethics and History of Medicine, Ruhr-University Bochum, Markstraße 258a, 44799 Bochum, Germany; 2 Diaconia University of Applied Sciences Bielefeld, Bethelweg 8, 33617 Bielefeld, Germany; 3 Institute for Ethics, Wilhelm Löhe University of Applied Science, Fürth, Germany; 4 Department of Medicine III, Klinikum der Universität München, Campus Großhadern, Munich, Germany

**Keywords:** Limiting treatment, End-of-life care, Medical ethics, Survey, Cross-sectional study

## Abstract

**Background:**

Limiting treatment forms part of practice in many fields of medicine. There is a scarcity of robust data from Germany. Therefore, in this paper, we report results of a survey among German physicians with a focus on frequencies, aspects of decision making and determinants of limiting treatment with expected or intended shortening of life.

**Methods:**

Postal survey among a random sample of physicians working in the area of five German state chambers of physicians using a modified version of the questionnaire of the EURELD Consortium. Information requested referred to the patients who died most recently within the last 12 months. Logistic regression was performed to analyse associations between characteristics of physicians and patients regarding limitation of treatment with expected or intended shortening of life.

**Results:**

As reported elsewhere, 734 physicians responded (response rate 36.9%) and of these, 174 (43.2%) reported a withholding and 144 (35.7%) a withdrawal of treatment. Eighty one physicians estimated that there was at least some shortening of life as a consequence. In 25.9% of these cases hastening death had been discussed with the patient at the time or immediately prior to this action. Types of treatment most frequently limited was artificial nutrition (*n* = 35). Bivariate analysis indicates that limitation of treatment with possible or intended shortening of life for patients aged > 75 years is performed significantly more often (*p* = 0.007, OR 1.848). There was significantly less limitation of treatment in patients who died from cancer compared to patients with other causes of death (*p* = 0.01, OR 0.486). There was no significant statistical association with physicians’ religion, palliative care qualification or frequencies of limiting treatment.

**Conclusions:**

In comparison to recent research from other European countries, limitation of treatment with expected or intended shortening of life is frequently performed amongst the investigated sample. The role of clinical and non-medical aspects possibly relevant for physicians’ decision about withholding or withdrawal of treatment with possible or intended shortening of life and reasons for non-involvement of patients should be explored in more detail by means of mixed method and interdisciplinary empirical-ethical analysis.

## Background

Limiting treatment, in the sense of withholding and/or withdrawal of medical measures, is part of clinical practice across different fields of medicine [1–5]. At the same time, there is considerable variation in frequency. More than a decade ago, the EURELD study, for example, showed a frequency of 4% in Italy, while physicians in Switzerland reported limitation of treatment in 28% of cases [6]. In addition, there have been changes observed over time with regard to the frequency of these decisions [1, 7, 8].

Although accepted in many jurisdictions, limitation of treatment is still challenging for physicians [9–11]. There is evidence that decisions about the intensity of treatment in patients near the end of life vary considerably and that these variations cannot be explained fully by medical factors [12–14]. Qualitative studies [15] and survey research suggest that physicians’ values and other non-medical factors contribute to the variation in practice [16–18].

While the practice of limiting treatment has been researched in several countries [1, 19, 20], there is a scarcity of robust data in Germany. Parts of the data gathered in Germany more recently are difficult to interpret due to the vague terminology used for capturing the different end-of-life practices [21]. Other studies are limited to particular clinical fields, such as palliative care, intensive care or oncology [3, 22–24]. Furthermore, some of the data on limitation of treatment near the end of life available were gathered almost two decades ago [25, 26]. In the light of the changes regarding the ethico-legal framework for decisions at the end of life [27], it is possible that the frequency of (some of the end-of-life practices) or reporting of the practice changes over time. This might be particularly the case given the fact that limitation of treatment with possible shortening of life has gained particular scientific and public interest in Germany in the course of the debate about legislating advance directives. While the German courts had confirmed patients’ right to reject treatment decades ago, legislation which confirms patients’ right to limit any treatment in advance has only been in existence since 2009.

The right of a patient to reject treatment is an important normative cornerstone. However, decisions about limiting treatment are often discussed in daily clinical practice in situations in which patients are open to more treatment, but in which the benefit and harm of specific treatment needs to be evaluated critically. In addition to the clinical challenge to determine benefit and harm in the light of frequent absence of evidence in such situations, there are also ethically relevant challenges. How far physicians can evaluate the benefit and harm of treatment without taking into account the subjective perspective of the patient, for example, is a matter for debate.

Data about frequencies and characteristics of decisions about limiting treatment and the decision-making process are important to identify challenges and to inform guidance on good clinical practice with regard to these decisions. The data which has been elicited in representative studies in various countries [1, 6–8, 28, 29] cannot necessarily be transferred to the situation in Germany. This is because country-specific, cultural and legal differences may not only influence the findings, but also the interpretation of findings regarding guidance on good clinical practice concerning limiting treatment.

The aim of this paper is to provide an in depth empirical analysis of practices of treatment limitation. We present findings on limiting treatment collected in a survey of physicians in five state chambers of physicians in Germany in 2013. The analysis focuses on those practices in which responding physicians expected or intended shortening of life. The empirical findings will be interpreted in the light of available international survey research and with reference to ethical and legal standards relevant for decisions about withholding or withdrawal of treatment in Germany.

## Methods

### Participants and mailing procedure

The authors conducted a postal cross-sectional survey on end-of-life practices among a random sample of 2,003 physicians from five German state chambers of physicians (Westphalia-Lippe, North Rhine, Saarland, Saxony and Thuringia), which cover around a third of all physicians working in Germany. The methods and first findings regarding the range of different end-of-life practices have been published elsewhere [30]. In line with the procedure approved by the Research Ethics Committee of the Medical Faculty of the Ruhr-University Bochum (AZ 4196–11) there was no identifying code on the questionnaire for the protection of anonymity. Physicians received the questionnaire and a leaflet with information on purpose, potential benefits and risks of the study as well as research procedure for the first time in the second calendar week of 2013. Consent was taken for granted when physicians returned the questionnaire anonymously. All physicians received a reminder and a second questionnaire in calendar week four, together with the information that only one questionnaire should be returned by each physician. Due to the procedure, it was not possible to conduct a non-responder analysis.

### Questionnaire

We used a modified version of the EURELD questionnaire, which had already been used in the German-speaking part of Switzerland [6] and in an earlier study on end-of-life practices of German palliative care physicians conducted by the second and last author of this paper, as the survey instrument [3, 22]. Changes that were made were distinctions of questions on actions from expected or intended effects [31] and three additional questions on physicians’ views of assisted suicide. Following the procedure described by Seale [31], potential participants had been informed on the first page of the questionnaire that all questions of the survey instrument refer to the patient who had most recently died under their care. Participants of the study who indicated on the cover page of the questionnaire that they had not cared for a dying patient within the last 12 months were asked to return the questionnaire with information only on their views on assisted suicide and socio-demographic aspects. Completed questionnaires were sent to a scientific institute for social research which recorded the data in a SPSS data file to avoid any direct contact between respondents and researchers. The relevant key questions can be found in Table 1.

**Table 1:** Key questions relevant for statistical analysis

(1) “Did you or another physician perform or did you make sure that one of the following actions would be performed: a) Withholding of treatment b) Withdrawing of treatment.”
(2) In case of withholding/withdrawing a treatment: “Did you or another physician assume that this action will probably or certainly hasten the death of the patient?”
(3) “Was death the consequence of withholding/withdrawing a treatment with the explicit intention to hasten death?”
Due to the structure of the questionnaire, we were only able to analyse the decision-making for the decisions on treatment limitation that were the limitations mentioned last and, therefore, the most important.

### Analyses

The raw data entry was double-checked within this institution. In addition, the plausibility of the data entered was checked by the first author. Free text comments regarding the type of treatment that had been limited were categorised by the first author together with the last author by means of a modified categorical system of different types of treatment which had been used in earlier research [3, 29]. Table 2 indicates the different steps of the analysis and the respective sample.

**Table 2:** Different steps of analysis and sample size

(1) Analysis of the whole sample regarding the frequency of limiting treatment as one aspect of end-of-life practices (*n* = 403)
(2) Analysis of the subgroup concerning “Types of limited treatment and expected consequences” (*n* = 104)
(3) Analysis of the subgroup “Decision-making and patient involvement” (*n* = 81)
(4) Regression analysis on determinants associated with limitation of treatment and expected shortening of life (*n* = 403)

The results of the descriptive analysis of end-of-life practices are provided as total numbers and valid percentages for the total sample or subgroups. Statistical analysis was performed with IBM SPSS Statistics version 23.0 for Windows. We explored associations between the limitation of medical treatment and characteristics from the side of the patient or the physician based on findings of earlier surveys [29, 32]. This included the possible influence of patients’ age [3, 33, 34], disease [3, 35], physicians’ religious affiliation [16] and specialisation in palliative care [3] with regard to limiting treatment with possible and/or intended shortening of life using binary logistic regression. Our hypotheses were as follows: a) treatment limitation with possible or expected shortening of life is performed more often in patients of older age; b) treatment limitation with possible or expected shortening of life is performed less often in patients dying from cancer; c) physicians who describe themselves as religious perform less treatment limitation with a possible or expected shortening of life than physicians who are non-religious; and d) physicians with a specialisation in palliative care perform treatment limitation with a possible or expected shortening of life more often than other medical specialists. Binary logistic regression was used to explore bivariate relationships between the dependent variable ‘treatment limitation with possible and/or intended shortening of life’ and four independent variables: (1) dichotomised patient’s age ≥ 75 years, (2) patient dying from cancer, (3) physician being religious, and (4) physician’s specialisation in palliative medicine. Odds ratios (ORs) and their 95% confidence intervals (CI) were computed. Subsequently, a multivariable logistic regression was performed with the aforementioned categories in one block using the enter method. *P*-values < 0.05 were considered significant.

## Results

As reported elsewhere [30], a sample of 734 respondents (response rate 36.9%) was obtained. A total of 403 physicians within this sample had cared for an adult patient who died within the 12 months prior to the survey. Of those physicians who had cared for a patient near the end of life, 219 (54.34%) reported a limitation of treatment. Of these, 174 (43.2%) reported a withholding and 144 (35.7%) a withdrawal of treatment (doctors could have both withheld and withdrawn treatment in the same patient). In the following, we report unpublished data of an in-depth analysis of determinants for limiting treatment and characteristics of the decision-making process.

### Types of limited treatment and expected consequences regarding shortening of life

Withholding or withdrawing of treatment with a possible or intended shortening of life was performed in 144/403 cases (35.7%). 135 physicians (33.5%) have performed a treatment limitation with a possible shortening of life and 19.1% (*n* = 77) with the explicit intention to shorten life (doctors could have had ambivalent intentions). As mentioned in the methods section and due to the structure of the questionnaire, details of decisions about withholding or withdrawal of treatment could only be further analysed if respondents indicated this type of end-of-life practice as the last in a row of several practices (e.g. symptom alleviation, assisted suicide) on the questionnaire. Accordingly, we could analyse in more detail the data of 75 patients for whom treatment limitation with *intended* shortening of life and 29 patients for whom limiting treatment with *possible* shortening of life (but no respective intention) was reported (step 2 of analysis; see method section). The characteristics of the 104 physicians who limited treatment and respective patient characteristics can be found in Tables 3 and 4.

**Table 3:** Characteristics of study participants *n* = 104

	*N* = 95	Percent
Medical specialty		
Internal medicine	25	26.3
General medicine	20	21.1
Anaesthesia	19	20.0
Surgery	17	17.9
Neurology/Psychiatry	7	7.4
Emergency medicine	2	2.1
Gynaecology	2	2.1
Otolaryngology	2	2.1
Urology	1	1.1
Missing data	9	--
Age		
< 36 years	29	27.9
36–45 years	29	27.9
46–55 years	24	23.1
56–65 years	17	16.3
> 65 years	5	4.8
Gender		
Male	64	61.5
Female	40	38.5
Religion		
Protestant	40	38.5
Catholic	39	37.5
No religion	19	18.3
Islamic	2	1.9
Other	4	3.8

**Table 4:** Patient characteristics *n* = 104

	Number	Percent
Age		
< 75 years	34	32.7
≥ 75 years	70	67.3
Gender		
Male	54	52.4
Female	49	47.6
Cause of death^a^		
Cancer	36	34.6
Cardiovascular disease	27	26.0
Disease of the nervous system	16	15.4
Respiratory disease	9	8.7
Other/Unknown	21	20.2

^a^multiple answers possible

Out of the subsample defined above, 41 physicians of the 104 respondents (40.6%) estimated the shortening of life as a consequence of limiting treatment to be between 1 and 7 days. A total of 20 physicians (19.8%) estimated that there was no shortening of life in the concrete patient as a consequence of their action. In ten cases (9.9%), shortening of life was estimated to be between 1 and 6 months. In this group, it was reported that six patients had died from cancer, two from cardiovascular diseases and two from other or unknown diseases. Table 5 summarises the findings on estimated shortening of life as a consequence of limiting treatment. The types of treatment limited most frequently were artificial nutrition (*n* = 35), antibiotics (*n* = 33) and the administration of catecholamines (*n* = 27). Table 6 summarises the data reported on types of treatments which had been withheld or withdrawn.

**Table 5:** Expected shortening of life reported by physicians who limited treatment

Action and consequence: Time of estimated shortening of life	Limiting treatment with intended shortening of life (*n* = 75)						Limiting treatment with possible shortening of life (*n* = 29)					
	Total		Withholding		Withdrawing		Total		Withholding		Withdrawing	
	*N*	%^a^	*N*	%	*N*	%	*N*	%^a^	*N*	%	*N*	%
1–6 months	10	13.7	6	12.5	4	16.0	--	--	--	--	--	--
1–4 weeks	14	19.2	8	16.7	6	24.0	3	10.7	2	13.3	1	7.7
1–7 days	32	43.8	22	45.8	10	40.0	9	32.1	7	46.7	2	15.4
<24 h	9	12.3	8	16.7	1	4.0	4	14.3	--	--	4	30.8
Not shortened at all	8	11.0	4	8.3	4	16.0	12	42.9	6	40.0	6	46.2
Missing data	2	--	2	--	--	--	1	--	--	--	1	--

^a^Percentages refer to valid responses (*n* = 73 for limiting treatment with intended shortening of life and *n* = 28 for limiting treatment with possible shortening of life)

**Table 6:** Number of patients (*n* = 104) and types of limited treatment which was withheld and/or withdrawn^a^

	Withholding of treatment		Withdrawal of treatment	
	(*n* = 87)		(*n* = 60)	
	*N*	%	*N*	%
Nutrition	22	25.3	13	21.7
Antibiotics	19	21.8	14	23.3
Catecholamines	15	17.2	12	20.0
Hydration	15	17.2	11	18.3
Dialysis	14	16.1	9	15.0
Respiration	14	16.1	5	8.3
Chemotherapy	12	13.8	8	13.3
Resuscitation	9	10.3	7	11.7
Medication^b^	8	9.2	15	25.0
Intubation	6	6.9	-	-
Other	4	4.6	2	3.3
Surgery	4	4.6	1	1.7
Diagnostic tests	3	3.4	-	-
Radiotherapy	2	2.3	1	1.7
Hospital admission	2	2.3	-	-
Transfusions	1	1.1	1	1.7

^a^multiple answers possible

^b^other than antibiotics, chemotherapy and catecholamines

### Decision-making and patient involvement

Eighty one physicians estimated that there was at least some shortening of life as a consequence of the limitation of treatment (see Table 5). This sample is a subgroup of the cases with intended or possible shortening of life (*n* = 104) reported above. A total of 21 of the physicians in this subsample (25.9%) reported that hastening death as a possible or intended consequence of limiting treatment had been discussed with the patient *at the time or immediately prior to* this action (see Table 7). In 23 cases (28.4%), the action was discussed with the patient *some time* before. In 37 cases (45.7%), the estimated hastening of death due to the limitation of treatment performed was *not discussed* with the patient *at all*. In 29 of these cases (78.4%), the patient was considered as not able to evaluate his/her situation and make an adequate decision about it at all by the physician.

**Table 7:** Questions concerning the end-of-life discussion

Has the possible or intended shortening of life been discussed with the patient?	Number	Percent
- Yes, at the time or shortly before this action	21	25.9
- Yes, some time before	23	28.4
At the time of discussion: Did you consider the patient able to evaluate his/her situation properly and to take a competent decision?		
- Yes, the patient was able to do so	34	77.3
- No, not entirely able to do so	8	18.2
- No, not able at all	2	4.5

In six cases (16.2%), the patient was judged as not entirely able to evaluate his/her situation. In two cases (5.4%), the patient was judged able to evaluate the situation properly. In one of these cases, “dementia” was given as a reason for not discussing hastening of death, in the other, no specific reason was indicated for not discussing limitation of treatment with hastening death as a possible consequence.

### Determinants associated with limitation of treatment and expected shortening of life

Based on our hypotheses (see method section), we investigated possible associations between patient disease (cancer versus non-cancer), age and physicians’ religious affiliation and specialisation in palliative medicine with frequencies of limiting treatment with shortening life. Bivariate analysis shows that age ≥ 75 years is significantly associated with limiting treatment with a possible and/or intended shortening of life (*p* = 0.007, OR 1.848, CI [1.183;2.886]). However, this association could not be affirmed in multivariable regression which included patient disease (cancer versus non-cancer), physicians’ religious affiliation and specialisation in palliative medicine (*p* = 0.205, OR 1.432, CI [0.822;2.496]). Compared to patients dying from other diseases, limitation of treatment at the end of life in patients dying from cancer was performed significantly less often (bivariate analysis: *p* = 0.000, OR 0.409, CI [0.261;0.64], multivariable analysis: *p* = 0.01, OR 0.486, CI [0.281;0.84]). There were no statistically significant differences regarding the performance of treatment limitation between physicians with and without religious affiliation (bivariate regression: *p* = 0.951, OR 0.984, CI [0.581;1.666], multivariable regression: *p* = 0.829, OR 1.072, CI [0.572;2.011]). There was also no significant association between the physician being specialised in palliative care and limiting treatment with possible or intended shortening of life (bivariate regression: *p* = 0.440, OR 0.742, CI [0.348;1.583], multivariable regression: *p* = 0.727, OR 0.866, CI [0.386;1.943]). Table 8 summarises the findings of bi- and multivariable logistic regression analysis regarding the association of socio-demographic factors of patients and physicians with a prevalence for limiting treatment with possible or intended shortening of life.

**Table 8:** Results of logistic regression on patients’/physicians’ characteristics and limiting treatment (*n* = 403)

	Treatment limitation with possible/intended shortening of life	
Socio-demographic factors	Bivariate logistic regression	Multivariable regression
Patient age ≥ 75 years	*p* = 0.007, OR 1.848, CI [1.183;2.886]	*p* = 0.205, OR 1.432, CI [0.822;2.496]
Patient dying from cancer	*p* = 0.000, OR 0.409, CI [0.261;0.64]	*p* = 0.01, OR 0.486, CI [0.281;0.84]
Physician being non-religious	*p* = 0.951, OR 0.984, CI [0.581;1.666]	*p* = 0.829, OR 1.072, CI [0.572;2.011]
Physician’s specialisation in palliative medicine	*p* = 0.440, OR 0.742, CI [0.348;1.583]	*p* = 0.727, OR 0.866, CI [0.386;1.943]

## Discussion

This paper provides in-depth analyses of practices and decision-making about limiting treatment of German physicians in those cases in which shortening of life was expected or even intended by the treating physician. The study contributes data elicited with an internationally widely used survey instrument [6, 8, 29] from a sample of German physicians who work in different clinical fields. In the following, we will compare the findings with data elicited in other countries and analyse the results against the background of current ethical and legal guidance in Germany.

### Frequency of limiting treatment

Practices of limitation of treatment with intended or possible shortening of life in our sample are the second most frequent decisions at the end of life (*n* = 144, 35.7%). Withholding or withdrawal of medical measures with intended or possible shortening of life takes place less often than alleviation of symptoms (*n* = 299, 86.7%), but more often than “palliative sedation” (*n* = 105, 30.8%) or the much discussed practices of physician-assisted suicide (*n* = 1, 0.3%) and euthanasia (*n* = 2, 0.6%) [30]. Given that the survey instrument, similar to the study from Seale [36], clearly distinguishes questions on the actual practice (e.g. withholding or withdrawing) from actions with expected consequences or the intention of the physician (i.e. shortening of life), we believe that the survey instrument avoids misunderstandings and that the figures gathered are robust [37, 38]. Compared to the study published by Seale in 2009 [36], treatment limitation with intended shortening of life (19.1 vs. 7.4%) as well as with possible shortening of life (33.5 vs. 28.9%) was practiced more frequently in our sample. Moreover, our figures are comparable to data published recently by Bosshard et al. in Switzerland [7] as well as the data presented by Onwuteaka et al. in the Netherlands [8]. Compared to the data published by Chambaere et al. [1] withholding and withdrawing of life-prolonging treatment was performed more frequently in our sample. However, the latter three studies do not distinguish between data about the practice (“limiting treatment”) and the practice combined with a consequence (“limiting treatment with possible shortening of life”) which means that comparison is limited. In addition, the differences in methodology such as the chosen approach to physicians based on death certificates signed by the respective physicians in the aforementioned three studies should be considered as a factor relevant for the reported differences.

Moreover, our results have to be interpreted with caution due to potential selection bias in a sample in which more than 60% of physicians did not respond. One explanation for the relatively high rate of limiting treatment may be an increased awareness of the possibility of limiting treatment lawfully in Germany if this is in accordance with the patients will. Debates around the legislation of advance directives (enacted) and the more recent debate about a law on assisted suicide (enacted in 2015) during which the normative framework for limiting treatment at the end of life has been reiterated in many scientific and popular articles may be triggers for such an increased awareness. In this respect the reported high numbers of limiting treatment could be interpreted as positive effects of the debate in the sense of an increased awareness amongst physicians that limiting treatment is an essential part of high quality care at the end of life.

### Decision-making and patient involvement

How far patients are involved in decisions about limiting treatment with associated shortening of life is of particular interest from a clinical-ethics perspective. This is because the normative evaluation of benefit and harm of a treatment against possible shortening of life is a matter which varies according to personal values and preferences. Accordingly, the involvement of patient in such evaluation can inform a decision which takes into account the patients’ personal stance. The responses of the physicians indicate that in about half of the cases, decisions about limiting and the consequence of possible or intended hastening death had been discussed with the patient at least some time prior to the actual limiting of treatment. Evidence indicates that the time of discussing end-of-life issues depends on perceived competences and further characteristics from the side of the physician [39, 40]. In the group in which there had not been a discussion with patients, this has been explained by the respondents with reference to the physicians’ judgment that the patient was deemed not able to make a competent decision. The high number of cases in which decisions about limiting treatment with shortening of life are being made on behalf of incompetent patients point to the importance of communicating options and preferences early, as this is advocated by proponents of advance care planning [41, 42] which has been recently incorporated as part of the German legislation on hospice and palliative care (Gesetz zur Verbesserung der Hospiz- und Palliativversorgung in Deutschland, 8.12.2015).

### Clinical and non-medical determinants in limiting treatment

The statistical analysis of determinants associated with decisions about limiting treatment with possible or intended shortening of life confirms, as part of the bivariate analysis, earlier findings that there is significantly more limiting of treatment with possible and/or intended shortening of life in older patients [3, 33, 34]. However, logistic regression also indicates that there are further factors possibly relevant to these decisions. The relatively high number of (younger) cancer patients in the sample in whom there is significantly less treatment limitation may be a confounding factor responsible for the fact that the association between age and treatment limitation could not be confirmed in the multivariable analysis. From a clinical perspective, a higher prevalence of treatment limitation in elderly patients is of little surprise, since older age is often associated with a worse health condition which restricts treatment options. Furthermore, there is a lack of evidence for the effects of treatment in elderly patients, since these patients are often underrepresented in clinical research [43]. However, quantitative and qualitative research raise questions whether “biological age”, in the sense of fitness, is the only reason for treating elderly patients less intensively. Findings from qualitative interviews with oncologists in Germany and the UK suggest that images or stereotypes of the situation of younger or elderly patients may influence decisions about offering or limiting cancer treatment [18, 44]. There may be a risk of both overtreatment of younger as well as undertreatment of older patients if health professionals do not reflect that the evaluation of life situations of younger or older patients may be value-laden. Our research did not confirm our hypotheses regarding the influence of the physicians’ religious affiliation or specialisation in palliative care with the frequency of this practice. Regarding the possible influences of the religion of the physician, our findings seem to be in contrast with those of Seale [16], who found that doctors who described themselves as non-religious more often made decisions with foreseen or intended ending of life. However, one explanation for this difference may be that our survey instrument did not explicitly investigate perceived religious attitudes, but only formal affiliation. With regard to the lack of an association between limiting treatment and physicians’ additional qualification in palliative care, one explanation is the low number of physicians in our sample who had such a qualification (*n* = 33) and the over-representation of patients with cancer.

### Limitations and strengths

The refusal of the majority of the state chambers of physicians to draw a random sample for this study and the low response rate of 36.9% are limitations with regard to the representativeness of the sample of physicians participating. Another limitation is the possible misunderstanding of single questions given the complex topic surveyed in this study [11]. Further limitations encompass socially desirable answers and recall bias. A strength of the study is the use of an internationally widely used instrument with a language which avoids misleading terms, such as “passive euthanasia”. Furthermore, the study is not limited to selected fields, but reports findings from across different clinical practices. Finally, the interpretation of data is a result of multidisciplinary work involving philosophers, health researchers and authors with clinical experience in end-of-life care.

## Conclusions

Limitation of treatment with expected and intended hastening death is, compared to international research, frequently practiced in this sample of German physicians. Clinical aspects (i.e. cancer) and socio-demographic characteristics on the side of patients, for instance, seem to influence decisions about limitation of treatment with associated shortening of life. To be able to understand how far these factors influence decisions about limiting treatment with associated shortening of life using an empirical mixed-method approach, it is important to combine empirical research with conceptual analysis rooted, for example, in ethics or sociology.

## Abbreviations

CI: Confidence interval
OR: Odds ratio
